# ELISA F29 –A therapeutic efficacy biomarker in Chagas disease: Evaluation in pediatric patients treated with nifurtimox and followed for 4 years post-treatment

**DOI:** 10.1371/journal.pntd.0011440

**Published:** 2023-06-23

**Authors:** Rocio Rivero, Mónica Inés Esteva, Erya Huang, Leylen Colmegna, Jaime Altcheh, Ulrike Grossmann, Andrés Mariano Ruiz, on behalf of the CHICO and CHICO SECURE Study Groups

**Affiliations:** 1 Instituto Nacional de Parasitología “Dr Mario Fatala Chaben” ANLIS MALBRÁN, Ministerio de Salud, Buenos Aires, Argentina; 2 Bayer US LLC, Whippany, New Jersey, United States; 3 LAT Research, Buenos Aires, Argentina; 4 Hospital de Niños Ricardo Gutiérrez and Instituto Multidisciplinario de Investigación en Patologías Pediátricas (IMIPP), CONICET, Buenos Aires, Argentina; 5 CONICET, Consejo Nacional de Investigaciones Científicas y Técnicas, Buenos Aires, Argentina; 6 Bayer AG, Research and Development Pharmaceuticals, Berlin, Germany; US Food and Drug Administration, UNITED STATES

## Abstract

**Background:**

Measurement of the success of antitrypanosomal treatment for Chagas disease is difficult, particularly in the chronic phase of the disease, because anti-*Trypanosoma cruzi* antibodies persist in serum for prolonged periods. We studied the effects of nifurtimox administered by two different treatment regimens on the *T*. *cruzi* calcium-binding flagellar protein F29 in children diagnosed with Chagas disease measured using an enzyme-linked immunosorbent assay (ELISA) technique (ELISA F29).

**Methods and principal findings:**

In a phase 3, randomized, double-blind, parallel-group, historically controlled study (ClinicalTrials.gov NCT02625974), blood samples obtained from children diagnosed with Chagas disease and treated with nifurtimox for either 60 days or 30 days were analyzed using an ELISA with an F29 recombinant protein as the antigen, as well as conventional serological tests (recombinant ELISA and indirect hemagglutination assay). In an exploratory approach, serological response to nifurtimox treatment was evaluated for 4 years post-treatment. In both treatment groups, the number of patients with negative ELISA F29 values increased over the period of observation. The incidence rate of negative seroconversion using ELISA F29 was 22.94% (95% CI: 19.65%, 26.63%) in the 60-day treatment group and 21.64% (95% CI: 17.21%, 26.86%) in the 30-day treatment group. In the subpopulation of patients who tested seropositive for F29 before nifurtimox treatment, 88 patients (67.7%) in the 60-day regimen and 39 patients (59.1%) in the 30-day regimen were F29 seronegative at 4 years post-treatment. All patients who had a positive ELISA F29 test at baseline and seroconverted to negative measured by conventional serology reached seronegativity in ELISA F29 earlier than in conventional serology.

**Conclusions:**

The results demonstrate a serological response to treatment with nifurtimox measured by the ELISA F29 test in children diagnosed with Chagas disease. The F29-based ELISA can be considered a potential early marker of response to antitrypanosomal therapy for Chagas disease.

**Trial registration:**

ClinicalTrials.gov NCT02625974.

## Author summary

Chagas disease (American trypanosomiasis) is a potentially life-threatening parasitic disease caused by *Trypanosoma cruzi*. Measurement of the success of antitrypanosomal treatment is difficult, particularly in the chronic phase of the disease, because serum anti-*T*. *cruzi* antibodies persist for prolonged periods even after complete elimination of the parasite. Thus, markers for early assessment of response to antitrypanosomal therapy could be useful tools to monitor response to drug treatment in Chagas disease. In this prospective study, we analyzed serum samples obtained from children diagnosed with Chagas disease and treated with nifurtimox (an antitrypanosomal drug with proven effectiveness). Before treatment and at each annual visit for the 4 years after treatment, we measured levels of antibodies to a specific antigenic protein (F29) of *T*. *cruzi* parasite using an established immunological technique; enzyme-linked immunosorbent assay (ELISA). The number of patients with negative ELISA F29 results increased over the period of observation after the end of treatment. At 4 years post-treatment, we found seronegative ELISA F29 test results in 67.7% of the patients in the 60-day nifurtimox regimen and in 59.1% of the patients in the 30-day nifurtimox regimen. The F29-based ELISA can be considered a useful method to monitor responses to drug treatment in children with asymptomatic Chagas disease.

## Introduction

Chagas disease (American trypanosomiasis) is a potentially life-threatening parasitic disease that for many years has been endemic in 21 Latin American countries and areas of southern USA [1]. Although the disease was once confined to the Americas, migration from endemic countries has led to the appearance of Chagas disease in nonendemic regions as well [2]. Currently, Chagas disease is predominantly urban, with at least two-thirds of infected people living in cities. Worldwide, it is estimated that only 10% of people who have Chagas disease are aware of their diagnosis. Consequently, there is a large number of individuals who, unaware of their infection, do not seek treatment and others who do not have access to adequate health care [3], which creates a major barrier for more effective control of the disease.

The protozoan *Trypanosoma cruzi*, which is the causal agent of the disease, is transmitted by triatomine bugs (“kissing bugs”) but also by either blood transfusion or organ transplantation from infected donors and from infected mothers to their children. The course of infection includes an acute phase that occurs shortly after becoming infected and, if untreated, a subsequent chronic phase; the latter is subdivided into symptomatic and asymptomatic forms. In the chronic symptomatic phase, approximately 30% of patients present clinical evidence of heart disease or megaviscera [4]. The majority of cases remain asymptomatic, although subtle disease-related changes are detectable [2], but infected for life.

The diagnostics recommended by national organizations and the Pan American Health Organization (PAHO) includes the combining of two positive serological tests: enzyme-linked immunosorbent assay (ELISA) and indirect hemagglutination assay (IHA) or indirect immunofluorescent assay (IFA), with potentially a third test if the results are conflicting [5]. The etiological treatment of Chagas disease is carried out using the antiparasitic drugs nifurtimox and benznidazole [6], which are indicated for the acute phase and for recent *T*. *cruzi* infections in children and adults. Measurement of the success of antitrypanosomal treatment in infected patients presents difficulties, particularly in the chronic phase of the disease. Because anti-*T*. *cruzi* antibodies persist long-term, even after treatment, conventional serology remains reactive many years after treatment and traditional parasitological methods do not have appropriate sensitivity. It can take years subsequent to treatment before patients with chronic Chagas disease become cured according to the current criterion of conversion of serological response to negative as measured by conventional assays, rendering measurement of treatment success lengthy [7]. This is a major challenge, especially for clinical studies in Chagas disease, and demonstrates the need to have sensitive and specific tools that allow the early monitoring of the efficacy of antitrypanosomal treatment in a safe and efficient manner [8].

Potential biomarkers of early therapeutic responses have been tested in different clinical contexts. An ELISA using a 29 kDa flagellar recombinant F29 antigen of the parasite (ELISA F29) was developed [9], which has been successfully assessed as an early biomarker of response to treatment with benznidazole in children [10] and in children and adults treated with antitrypanosomal therapy [11,12]. Significant differences in the reactivity towards F29 were observed between treated and untreated patients, and ELISA F29 is proposed as a useful method to monitor the response to drug treatment [8].

In a prospective, historically controlled, phase 3 clinical trial, the CHagas disease In Children treated with nifurtimOx follow-up for SEroconversion and CURE (CHICO and CHICO SECURE) trial, we followed pediatric patients with Chagas disease over a period of 4 years after treatment with a new formulation of nifurtimox and evaluated the incidence rate of seronegative conversion and their serological response to nifurtimox treatment using conventional serological methods (recombinant ELISA, total purified antigen ELISA, and IHA) and ELISA F29 at annual follow-ups. The progress of seronegative conversion detected by ELISA F29 and conventional serological methods during follow-up was compared. The results obtained for conventional serology, parasitological tests, and safety, as well as for ELISA F29 at first year of follow-up, are reported elsewhere [13].

## Methods

### Ethics statement

The study was approved by the respective independent ethics committees of participating investigational sites (Argentina: Comité de Ética, Hospital General de Agudos J. A. Fernández, Buenos Aires; Comité de Ética Independiente en Investigación Clínica "Dr. Carlos A. Barclay", Buenos Aires; Comité Institucional de Revisión de Protocolos de Investigación, Hospital de Niños Sor María Ludovica, La Plata; Comité de Ética en Investigación del Hospital de Niños Ricardo Gutiérrez, Buenos Aires; Comité de Ética en Investigación, San Miguel de Tucumán; Comité de Ética en Investigación del Hospital General de Niños, Buenos Aires; Comité de Ética en Investigación Hospital FJ Muñiz, Buenos Aires; Comité Independiente de Ética Médica del Noroeste Argentino, San Miguel de Tucumán; Comité de Ética del Hospital Pediátrico Dr Humberto Notti, Mendoza; Comité de Bioética, Hospital Dr Fernando Barreyro, Posadas. Colombia: Comité de Ética en Investigaciones de la Fundación Cardiovascular de Colombia, Floridablanca; Centro de Atención e Investigación Médica CAIMED S.A., Bogota; Comité de Ética en Investigación Hospital Mental Antioquia, Bello; Universidad del Norte, Barranquilla; Bolivia: Comité de Ética de CEADES, Cochabamba; Comité de Ética Hospital Manuel Ascencio Villarroel, Punata). Written informed consent of the patient and/or their parent(s) or legally authorised representative(s) was obtained prior to screening according to age and local regulations. In addition, depending on their age, patient assent was obtained if required by locally applicable laws and regulations in each country.

### Study design and procedures

In a phase 3, randomized, double-blind, parallel-group, historically controlled study (ClinicalTrials.gov NCT02625974), blood samples obtained from children diagnosed with Chagas disease were analyzed using ELISA F29. Blood samples were obtained for ELISA F29 immediately before nifurtimox treatment (baseline), on Days 7 (±1), 30 (±3), and 60 (±3) after the start of treatment and on Months 6 (±7 days), 12 (±7 days), 24 (±6 weeks), 36 (±6 weeks), and 48 (±6 weeks) after end of treatment.

Details of the study design and outcomes have been published elsewhere [13,14]. In brief, 330 pediatric patients aged from birth to less than 18 years of age with a diagnosis of Chagas disease were recruited at 25 hospital sites and randomized 2:1 to the nifurtimox 60-day or 30-day treatment regimens. Nifurtimox 30 mg and 120 mg tablets (Bayer HealthCare AG, Germany) were administered in three divided doses taken with food at 10–20 mg/kg/day for patients <12 years of age and of body weight <40 kg, and at 8–10 mg/kg/day for patients ≥12 years of age and of body weight ≥40 kg. A total of 295 pediatric patients enrolled at 17 sites in Argentina (n = 161), three sites in Bolivia (n = 54), and three sites in Colombia (n = 80) were followed for 4 years following nifurtimox treatment (CHICO and CHICO SECURE studies). Although the sample size of the study was based on the primary measure of the effectiveness of nifurtimox by conventional serological tests [13,14], it is considered informative for ELISA F29.Written informed consent of the patient and/or their parent(s) or legally authorized representative(s) was obtained prior to screening according to age and local regulations. In addition, depending on their age, patient assent was obtained if required by locally applicable laws and regulations in each country.

The design and all aspects of the conduct, evaluation, and documentation of the study conformed with good clinical practice guidelines and the guiding principles of the current version of the Declaration of Helsinki. The study also complied with applicable local law(s) and regulation(s), and all information identifying patients was collected, stored and analyzed in strict confidence and in accordance with local data protection laws.

### ELISA F29 laboratory test

An ELISA containing a recombinant 29 kDa *T*. *cruzi* calcium-binding protein as antigen was used to analyze serum samples taken from pediatric patients, as previously described [11]. The purified protein was resuspended in carbonate buffer pH 9.6 at a concentration of 5 μg/ml and dispensed to 96-well polystyrene plates at a final volume of 50 μl per well and fixed overnight at 4°C. Plates were blocked with phosphate-buffered saline (PBS) and 5% milk for 1 hour at room temperature. Dilution of the sera was performed in PBS and 1% milk at a concentration of 1/200. Incubation of sera was performed for 1 hour at 37°C. After 3 washes with PBS-Tween 0.05%, it was incubated with the second antibody: anti-human immunoglobulin G (IgG) coupled with peroxidase (Invitrogen Life Technologies, Frederick, MD, USA). After washing with PBS-Tween 0.05%, developing was performed with O-phenylenediamine dihydrochloride (OPD) 0.4 mg/mL in a citrate buffer pH 5 with 0.024% hydrogen peroxide (H_2_O_2_). Optical density (OD) at 490 nm was measured in a microplate reader (Mindray, Shenzhen, PR China).

### Outcomes

The serological response using ELISA F29 over time and the incidence rate of seronegative conversion measured by ELISA F29 were evaluated after treatment with nifurtimox. Furthermore, the time at which seronegative conversion was detected was compared for ELISA F29 and conventional serological methods: recombinant ELISA, total purified antigen ELISA, and IHA.

### Statistical analyses

Analysis of patient characteristics used the full analysis set (FAS), which included all patients who received at least one dose of study drug. For analysis of ELISA F29, a subpopulation was defined that included all patients in the FAS who had positive ELISA F29 results at baseline.

The incidence rate of seronegative conversion measured and confirmed by ELISA F29 in patients from the FAS was calculated. Incidence rate is the number of new cases of seronegative conversion over the study period, i.e. 4 years after end of nifurtimox treatment, divided by the total number of person-years, which was the estimate of the actual number of at-risk years for all patients who contributed to the study. Patients who used antitrypanosomal drugs other than the study drug before they were seroconverted to negative were not considered as seroconverted, but their time of observation until the first use of antitrypanosomal drugs was factored into the person-time calculation.

All analyses were descriptive, and no formal testing was performed. All statistical analyses were performed using Statistical Analysis System version 9.2 or higher (SAS Institute, Cary, NC, USA).

## Results

### Patient characteristics

Among the 330 patients randomly assigned to nifurtimox treatment for 60 days or 30 days, 318 completed part 1 of the study (CHICO) [13], of whom 295 (n = 197 in the 60-day treatment group and n = 98 in the 30-day treatment group) were followed for 4 years post-treatment in part 2 of the study (CHICO SECURE) [14]. All patients were diagnosed with Chagas disease according to the predefined inclusion criteria [13].

Among the 295 patients who were followed for 4 years post-treatment, 53.2% were females and 46.8% were males. The median age was 8.5 years (interquartile range: 2–13 years). About two-thirds of the patients were aged between either >6 and ≤12 years (37.3%) or >12 and <18 years (33.6%) at randomization (Table 1).

**Table 1:** Patient characteristics at randomization (FAS).

Characteristic	Nifurtimox 60-day regimen (n = 197)	Nifurtimox 30-day regimen (n = 98)	Total (N = 295)
Sex, n (%)			
Male	89 (45.2)	49 (50.0)	138 (46.8)
Female	108 (54.8)	49 (50.0)	157 (53.2)
Age group, n (%)			
≤2 years	30 (15.2)	13 (13.2)	43 (14.6)
>2 years to ≤6 years	31 (15.7)	12 (12.2)	43 (14.6)
>6 years to ≤12 years	73 (37.1)	37 (37.8)	110 (37.3)
>12 years to <18 years	63 (32.0)	36 (36.7)	99 (33.6)

### ELISA F29

In both treatment groups, the number of patients with negative ELISA F29 results increased over the period of observation after the end of nifurtimox treatment. At the 4-year follow-up the proportion of patients with seronegative ELISA F29 values was 77.2% in the 60-day regimen and 66.3% in the 30-day regimen (Table 2). The incidence rate of negative seroconversion using ELISA F29 was 22.94% (95% CI: 19.65%, 26.63%) in the 60-day treatment group and 21.64% (95% CI: 17.21%, 26.86%) in the 30-day treatment group.

**Table 2:** Serological responses to 60-day and 30-day nifurtimox treatment using ELISA F29 over the study period (FAS).

Time point of evaluation	Serological response, n (%)								
	Nonreactive			Reactive			Missing		
	60-day nifurtimox (n = 197)	30-day nifurtimox (n = 98)	Total (N = 295)	60-day nifurtimox (n = 197)	30-day nifurtimox (n = 98)	Total (N = 295)	60-day nifurtimox (n = 197)	30-day nifurtimox (n = 98)	Total (N = 295)
Baseline	67 (34.01)	32 (32.65)	99 (33.56)	130 (65.99)	66 (67.35)	196 (66.44)	0	0	0
1 year post-treatment	104 (52.79)	50 (51.02)	154 (52.20)	91 (46.19)	48 (48.98)	139 (47.12)	2 (1.02)	0	2 (0.68)
2 years post-treatment	141 (71.57)	62 (63.27)	203 (68.81)	47 (23.86)	30 (30.61)	77 (26.10)	9 (4.57)	6 (6.12)	15 (5.08)
3 years post-treatment	134 (68.02)	57 (58.16)	191 (64.75)	41 (20.81)	27 (27.55)	68 (23.05)	22 (11.17)	14 (14.29)	36 (12.20)
4 years post-treatment	152 (77.16)	65 (66.33)	217 (73.56)	38 (19.29)	25 (25.51)	63 (21.36)	7 (3.55)	8 (8.16)	15 (5.08)

**Fig 1:**
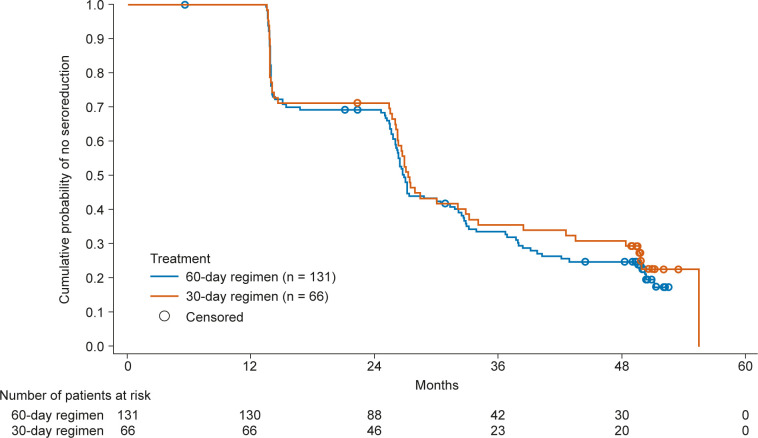
Kaplan-Meier survival curve of seronegative conversion in patients with positive ELISA F29 results at baseline.

A total of 99 of the 295 patients (33.6%) showed negative ELISA F29 values at baseline, and the proportion of such patients was similar in the two nifurtimox treatment groups (60-day regimen: 67 patients [34.0%]; 30-day regimen: 32 patients [32.7%]) (Table 2). All of these patients were diagnosed with Chagas disease according to the inclusion criteria [13] and most of these patients remained F29 antigen-negative at subsequent evaluations. To better assess the effect of nifurtimox, a subpopulation was selected consisting of all patients with positive ELISA F29 results at baseline (n = 196). In this subgroup, 88 patients (67.7%) in the 60-day regimen and 39 patients (59.1%) in the 30-day regimen were seronegative for the F29 antigen at 4 years post-treatment (Table 3 and Fig 1).

**Table 3:** Serological responses to 60-day and 30-day nifurtimox treatment using ELISA F29 over the study period (subpopulation of FAS*).

Time point of evaluation	Serological response, n (%)								
	Nonreactive			Reactive			Missing		
	60-day nifurtimox (n = 130)	30-day nifurtimox (n = 66)	Total (N = 196)	60-day nifurtimox (n = 130)	30-day nifurtimox (n = 66)	Total (N = 196)	60-day nifurtimox (n = 130)	30-day nifurtimox (n = 66)	Total (N = 196)
1 year post-treatment	40 (30.77)	19 (28.79)	59 (30.10)	89 (68.46)	47 (71.21)	136 (69.39)	1 (0.77)	0	1 (0.51)
2 years post-treatment	78 (60.00)	35 (53.03)	113 (57.65)	45 (34.62)	27 (40.91)	72 (36.73)	7 (5.38)	4 (6.06)	11 (5.61)
3 years post-treatment	78 (60.00)	32 (48.48)	110 (56.12)	37 (28.46)	24 (36.36)	61 (31.12)	15 (11.54)	10 (15.15)	25 (12.76)
4 years post-treatment	88 (67.69)	39 (59.09)	127 (64.80)	35 (26.92)	22 (33.33)	57 (29.08)	7 (5.38)	5 (7.58)	12 (6.12)

*The subpopulation of FAS consists of all patients with positive ELISA F29 results at baseline.

### Comparison of serological response to nifurtimox treatment measured by ELISA F29 and by conventional serology

**Fig 2:**
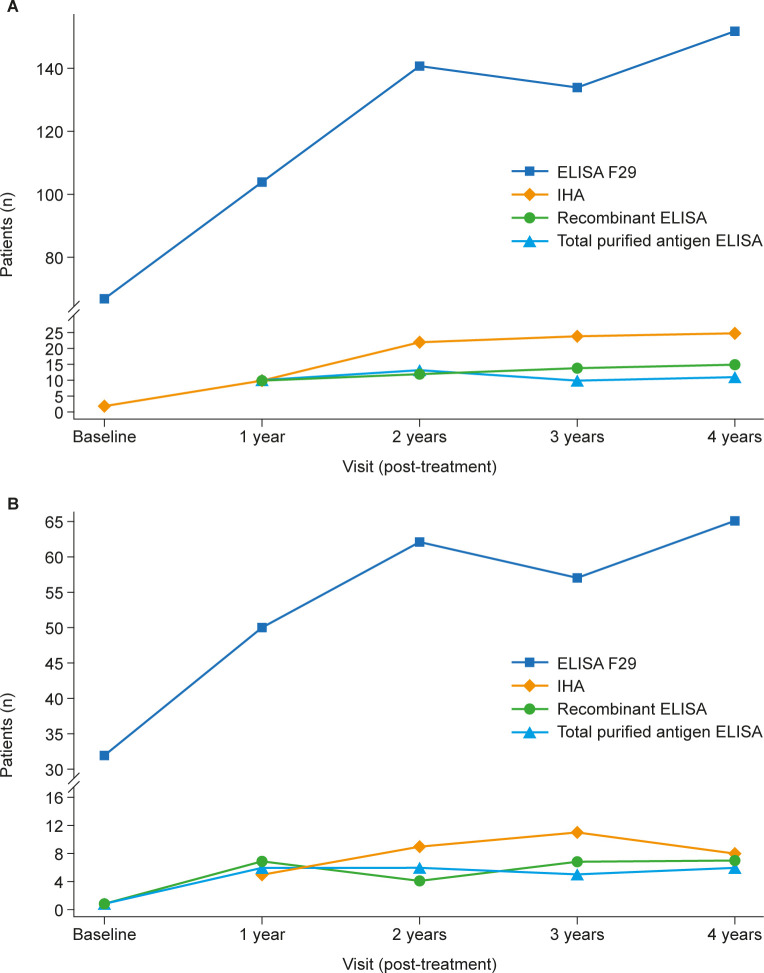
Number of patients with nonreactive values measured by ELISA F29, recombinant ELISA, total purified antigen ELISA, and IHA in the 60-day (A) and 30-day (B) nifurtimox treatment groups over the study period (FAS).

At the 4-year follow-up, the number of patients with seronegative ELISA F29 values was higher in both treatment groups compared with the number of patients with seronegative values measured by either recombinant ELISA, total purified antigen ELISA, or IHA (Fig 2). The incidence rate of negative seroconversion using ELISA F29 was also higher in both treatment groups compared with the rate measured by conventional serological assays (2.12% [95% CI: 1.21–3.45%] in the 60-day treatment group and 2.11% [95% CI: 0.91–4.16%] in the 30-day treatment group). All patients who had a positive ELISA F29 test result at baseline and seroconverted to negative by conventional serology tests reached seronegativity in ELISA F29 earlier than in conventional serology. However, no clear temporal relationship between the change in these two serological parameters was apparent. Details of the results using conventional assays have been published elsewhere [13,14].

## Discussion

Until now, there has not been an adequate parameter that reflects the early response or failure of etiological treatment for Chagas disease. Currently, the evaluation of the negativization of serology demonstrated by more than one type of conventional assay is the accepted criterion to establish successful treatment for Chagas disease [5,15]. In patients treated in the acute phase of Chagas disease, the efficacy of antitrypanosomal treatment can be demonstrated in a relatively short time because parasitemia becomes negative within or at the end of treatment or, at the latest, a few days after the end of treatment, and antibodies disappear completely in the majority of cases within 18 months of follow-up post-treatment [16–18]. In contrast, it has been reported that in 43% of adults with chronic Chagas disease, 27 years of post-treatment follow-up is necessary to facilitate nonreactive conventional serology test results [19]. Therefore, the lack of validated biomarkers of the early response to antitrypanosomal treatment in Chagas disease is a major challenge in the assessment of therapeutic efficacy of current and potential new treatments.

In this study, an ELISA containing a recombinant protein of the parasite (F29) was used to evaluate the serological profile in pediatric patients treated with nifurtimox and followed for 4 years post-treatment. The results clearly demonstrate conversion of ELISA F29 values to negative after antitrypanosomal therapy with nifurtimox in pediatric patients with acute and chronic Chagas disease. Similar results were observed in several previous clinical studies. In a retrospective cross-sectional study, for example, serum samples from patients with chronic Chagas disease (n = 118) followed for up to 23 years and either treated with trypanocidal therapy (nifurtimox or benznidazole) or left untreated were evaluated by ELISA F29, and its relation to conventional serology, blood parasite levels evaluated by xenodiagnosis, and clinical aspects were analyzed [11]. ELISA F29 was nonreactive in all patients who showed negative conventional serology after treatment (n = 30), in 82.4% of treated patients but who remained reactive by conventional serology (n = 34), and in 13% of the untreated patients (n = 54). Interestingly, all infected patients presenting electrocardiographic alterations compatible with chronic Chagasic cardiomyopathy were reactive to ELISA F29. Furthermore, patients with positive xenodiagnosis showed high reactivity with ELISA F29. A previous randomized, double-blind study of treatment with benznidazole compared with placebo in adult patients with chronic Chagas disease [20] found serological negativity revealed by ELISA F29 in 23.3% compared with 22.5% by conventional ELISA after one year of post-treatment follow-up. ELISA F29 was shown to be a stronger predictor of treatment success than the conventional serological test in these adult patients. In children aged 6–12 years of age diagnosed with Chagas disease and treated with benznidazole in a double-blind, randomized, placebo-controlled study, 62.1% of treated patients tested negative by ELISA F29 at their 4-year follow-up [10]. In our study, we also observed an overall increase in seronegative ELISA F29 results throughout the duration of post-treatment observation in our study of children with Chagas disease treated with nifurtimox for 60 days, which is the approved treatment duration in such patients; 67.7% of those who tested positive for F29 at baseline showed seronegative ELISA F29 results at 4 years post-treatment. Notably in the present study, ELISA F29 was nonreactive in all patients who seroconverted to negative based on conventional serological tests, and seronegativity in ELISA F29 occurred earlier than in conventional serology. The results demonstrate the usefulness of ELISA F29 as an early biomarker of treatment efficiency in children with Chagas disease.

It is known that different serological tests used to identify anti-*T*. *cruzi* antibodies can demonstrate discrepant results that are often attributed to antigenic differences among recombinant proteins or *T*. *cruzi* discrete typing units. About one-third of the children in our study showed negative ELISA F29 results at baseline and remained negative during the follow-up. Since diagnosis of Chagas disease in all study participants was confirmed at enrollment by conventional ELISAs and the ELISA F29 test is not considered a diagnostic assay, this observation could possibly represent false negatives; the proportion showing F29-negativity is, however, consistent with previous observations in a benznidazole study [21]. Other markers used in Chagas disease may show negative test results at baseline in some subjects. For example, the sensitivity of quantitative polymerase chain reaction (qPCR) tests for detecting *T*. *cruzi* deoxyribonucleic acid in blood samples varies and was shown to be highest for immunosuppressed heart transplanted patients (100% [25–75 percentiles = 100–100]), but lower for asymptomatic (56.5% [25–75 percentiles = 39.1–66.3%]) and for symptomatic (57.1% [25–75 percentiles = 14–75%]) patients with chronic Chagas disease [22]. Consistent with these findings, 46.8% of all patients in CHICO SECURE (45.2% in the 60-day regimen group and 50.0% in the 30-day regimen group) had negative PCR results at baseline [14].

In contrast to tests using a single antigen, such as the ELISA F29, the sensitivity of assays that include multiple antigens is higher, as recently shown in a multiplex serological approach [23]. In previous investigations, the multiplex serological approach was used for the detection of different specific antibodies against *T*. *cruzi* in the sera of patients with Chagas disease [24] and as a predictive monitoring tool to assess parasitological cure in children treated with antitrypanosomal therapy [23]. In comparison, the ELISA F29 is considered exclusively as an early biomarker for response to antitrypanosomal treatment in Chagas disease. On the other hand, it is considered difficult to find specific antigens for each stage of the parasite’s life cycle, which also points to the limitations of the tests currently used to assess cure and emphasizes the need for novel and improved therapeutic response markers. Based on a recently published target product profile for a test for the early assessment of treatment efficacy in Chagas disease patients, assay performance can be considered acceptable if showing a diagnostic sensitivity of 60% or better and a diagnostic specificity of at least 90% [25]. The presence of biomarkers in different pathologies represents an important tool for monitoring the disease and the clinical response to therapeutic treatment. In many cases, however, the prevalence of a biomarker depends on several factors that have not so far been fully elucidated. The presence of a sensitive marker thus constitutes an important opportunity to enhance understanding of the disease, as well as to monitor the effects of treatment.

In conclusion, our results demonstrate a serological response to treatment with nifurtimox, an established antitrypanosomal drug, evidenced by a decrease in anti-F29 antibodies in children diagnosed with Chagas disease. Our results are consistent with previous studies showing that ELISA F29 is a reliable and appropriate biomarker to assess response to antitrypanosomal therapy in Chagas disease. The earlier disappearance of antibodies against F29 relative to those detected in conventional ELISAs indicates variation within the overall serological response to antitrypanosomal treatment, and may be indicative of the subsequent elimination of the parasite.

## Supporting information

S1 Appendix Study site principal investigators of the CHICO and CHICO SECURE Study Groups. Investigators participated in both studies unless indicated otherwise. (DOCX)
