# Supplementary material for: ELISA F29 –A therapeutic efficacy biomarker in Chagas disease: Evaluation in pediatric patients treated with nifurtimox and followed for 4 years post-treatment
Source: PLoS Negl Trop Dis. 2023 Jun 23;17(6):e0011440. doi: 10.1371/journal.pntd.0011440 (PMC10325040; doi:10.1371/journal.pntd.0011440)
Supplement: S1 Appendix — (DOCX) [file pntd.0011440.s001.docx]

# Supporting information

**S1 Appendix** Study site principal investigators of the CHICO and CHICO SECURE Study Groups. Investigators participated in both studies unless indicated otherwise.

**Argentina**

H Abate MD, Hospital Pediátrico Dr Humberto Notti, Av Bandera de Los Andes 2603, M5519 Mendoza, Argentina (CHICO Study only)

L Arce MD, Hospital Pediátrico Dr Fernando Barreyro, Mariano Moreno 110, 3300 Posadas, Argentina

A Cancellara MD, Hospital General de Niños Dr Pedro de Elizalde, Montes de Oca 40, C1270AAN Buenos Aires, Argentina

A Caruso MD, Hospital de Niños Dr Héctor Quintana, José Hernández 624, 4600 San Salvador de Jujuy, Argentina

R de la Fuente MD, Hospital Papa Francisco, Barrio Solidaridad, Etapa 4, Manzana 405B, 4400 Salta, Argentina

M Díaz Ariza MD, Centro Integral Médico Actitud, B° Faldeo del Velasco Sur, Manzana D, Casa 3 La Rioja, Argentina

C Domínguez MD, Hospital Lagomaggiore, Timoteo Gordillo S/N, 5500 Mendoza, Argentina

R Duarte MD, Sanatorio San Juan, San Juan 975, W3400CBI Corrientes, Argentina

G Ensinck MD, Hospial de Niños Victor J Vilela, Virasoro 1855, 2000 Rosario, Argentina

A Falaschi MD, Hospital Pediátrico Dr Humberto Notti, Av Bandera de Los Andes 2603, 5519 Mendoza, Argentina

M Jofre MD, Vacunatorio Provincial San Juan, Bartolomé Mitre 344 Oeste, 5400 San Juan, Argentina

C Llapur MD, Hospital de Clínicas Presidente Dr Nicolás Avellaneda, Catamarca 2000, 4000 Tucumán, Argentina

I Menna MD, Hospital Fernández, Cerviño 3354, C1425AGP Buenos Aires, Argentina (CHICO SECURE Study only)

C Monla MD, Hospital Público Materno Infantil, Av Sarmiento 1301, 4400 Salta, Argentina

G Moscatelli MD, Hospital de Niños Ricardo Gutiérrez, Gallo 1330, C1425EFD Buenos Aires, Argentina

J Morales MD, Hospital de Niños Sor María Ludovica, Calle 14 no 1631 entre 65 y 66, 1900 La Plata, Argentina

T Ramirez MD, Centro de Enfermedad de Chagas y Patologias Regionales, Av. Belgrano Norte 660, Santiago del Estero, Argentina

A Romano MD, Instituto de Diagnóstico e Investigaciones Médicas de Formos, Jujuy 572, 3600 Formosa, Argentina

M Serjan MD, Hospital Fernández, Cerviño 3354, C1425AGP Buenos Aires, Argentina

M Sosa MD, Hospital de Enfermedades Infecciosas Dr F J Muñiz, Av Vélez Sarsfield 405, 1281 Buenos Aires, Argentina

**Colombia**

L Castro MD, Centro de Atención e Investigación Médica CAIMED Yopal, Calle 17 No. 26-05, Los Helechos, 0 Yopal, Colombia

J Dib MD, Centro Ensayos Clínicos - Fundación Salud para el Trópico, Km 21 Troncal del Caribe, 0 Santa Marta, Colombia

J Dib MD, Fundación Hospital Universidad del Norte, Calle 30 Autopista al Aeropuerto al lado del parque Muvdi- Soledad/Atlantico, Barranquilla, Colombia

F Quiroz MD, Fundación Cardiovascular de Colombia, Calle 155A N. 23-58, Piso 1A Urbanización El Bosque Sector EI, Floridablanca, Colombia

V Sierra MD, Centro de Atención e Investigación Médica CAIMED Yopal, Calle 17 No. 26 05, Los Helechos, 0 Yopal, Colombia (CHICO SECURE Study only)

**Bolivia**

L Ortiz Daza MD, Universidad Autónoma Juan Misael Saracho - Plataforma de Chagas – Tarija, España esquina Pasaje California, El Tejar, Tarija, Bolivia

J Pinto MD, Fundación CEADES Plataforma de Chagas Cochabamba, Av. Aniceto Arce y Oquendo, Cochabamba, Bolivia

J Sanchez MD, Fundación CEADES Hospital Manuel Ascencio Villarroel de Punata, Calle Gral. Achá 632 Punata, Bolivia
